# Supplementary material for: Mathematical Modeling of Vaccines That Prevent SARS-CoV-2 Transmission
Source: Viruses. 2021 Sep 24;13(10):1921. doi: 10.3390/v13101921 (PMC8539635; doi:10.3390/v13101921)
Supplement: Supplementary file 1 [file viruses-13-01921-s001.zip › viruses-1187776-supplementary.pdf]

Supplementary materials.

King County transmission model. Our mathematical model used in *section 3*.1-3.7 consists of a series of differential equations (*1*), which describe a series of compartments described in **Figure S2** and in the **Results.** In the equations, the subscript *i* denotes age-group and *j* denotes viral variant. Equations are listed here:

$$\frac{dS_i}{dt} = -\sum_{i}^{j} \lambda_{ij} S_i - \frac{v_i S_i}{S_i + R_i}$$

$$\frac{dE_{ij}}{dt} = \lambda_{ij} S_i - \gamma_1 E_{ij}$$

$$\frac{dA_{ij}}{dt} = (1 - p_i)\gamma_1 E_{ij} - (r_1 + d_A)A_{ij}$$

$$\frac{dDA_{ij}}{dt} = d_A A_{ij} - r_1 DA_{ij}$$

$$\frac{dP_{ij}}{dt} = p_i \gamma_1 E_{ij} - (\gamma_2 + d_P)P_{ij}$$

$$\frac{dI_{ij}}{dt} = \gamma_2 P_{ij} - d_i(t) I_{ij} - h_i^* I_{ij} - r_i^* I_{ij}$$

$$\frac{dD_{ij}}{dt} = d_P P_{ij} + d_i(t) I_{ij} - h_i^* D_{ij} - r_i^* D_{ij} - nf_i^* D_{ij}$$

$$\frac{dH_{ij}}{dt} = h_i^* I_{ij} + h_i^* D_{ij} - r_3 H_{ij} - f_i^* H_{ij}$$

$$\frac{dF_{ij}}{dt} = nf_i^* D_{ij} + f_i^* H_{ij}$$

$$\frac{dR_i}{dt} = \sum_{i}^{j} (r_1 A_{ij} + r_1 DA_{ij} + r_i^* \; I_{ij} + r_i^* D_{ij} + r_3 H_{ij}) - \frac{v_i R_i}{S_i + R_i}$$

$$\frac{dV_i}{dt} = -(1 - VE_{SUSC}) \sum_{i}^{j} \lambda_{ij} V_i + \frac{v_i S_i}{S_i + R_i}$$

$$\frac{dEV_{ij}}{dt} = (1 - VE_{SUSC}) \sum_{i}^{j} \lambda_{ij} V_i - \gamma_1 EV_{ij}$$

$$\frac{dAV_{ij}}{dt} = (1 - p_i + VE_{SYMP} p_i)\gamma_1 EV_{ij} - (r_1 + d_A)AV_{ij}$$

$$\frac{dDAV_{ij}}{dt} = d_A AV_{ij} - r_1 DAV_{ij}$$

$$\frac{dPV_{ij}}{dt} = (1 - VE_{SYMP}) p_i \gamma_1 EV_{ij} - (\gamma_2 + d_P)PV_{ij}$$

$$\frac{dIV_{ij}}{dt} = \gamma_2 PV_{ij} - d_i(t)IV_{ij} - h_i^* IV_{ij} - r_i^* IV_{ij}$$
$$\frac{dDV_{ij}}{dt} = d_P PV_{ij} + d_i(t)IV_{ij} - h_i^* DV_{ij} - r_i^* DV_{ij} - nf_i \;\; DV_{ij}$$
$$\frac{dHV_{ij}}{dt} = h_i^* IV_{ij} + h_i^* DV_{ij} - r_3 HV_{ij} - f_i \;\; HV_{ij}$$
$$\frac{dFV_{ij}}{dt} = nf_i^* DV_{ij} + f_i^* HV_{ij}$$
$$\frac{dRV_{ij}}{dt} = \sum_i^j (r_1 AV_{ij} + r_1 DAV_{ij} + r_{ij}^* \;\; IV_{ij} + r_i^* DV_{ij} + r_3 HV_{ij}) + \frac{v_i R_i}{S_i + R_i}$$

King County model parameters. Model parameters are listed as follows with values listed in the **Supplementary Table S1:**

p_i – proportion of the infections which become symptomatic by age in absence of a vaccine

γ_1, γ_2- progression rates from exposed (E) to infectious (A and P) to symptomatic (I)

h_i – hospitalization rate among severe cases by age in absence of a vaccine

h_i^* – hospitalization rate among diagnosed by age (calculated)

$r_{1\text{-}3}$ - recovery rate of the asymptomatic, mild symptomatic and hospitalized cases

r_i^* - recovery rate of the diagnosed symptomatic cases by age (calculated)

f_i^* – fatality rate among hospitalized by age

v_i – vaccination rate by age including prioritization strategy

VE_{SUSC} – vaccine efficacy in reducing the risk of symptomatic infection upon acquisition

VE_{SYMP}– vaccine efficacy in reducing the risk of hospitalization upon symptomatic infection

nf_i^*– death rate without hospitalization by age.

d_i – diagnostic rate by age.

The diagnostic rates are estimated using testing data from the Washington State Department of Health (DOH). The equation uses the average daily tests for the time period in

question divided by an estimate of the number of people desiring tests. The rates are divided across age groups according to the fraction of tests in each age group during that month. For tests, we use average daily tests by age-group as calculated monthly using a 7-day average taken mid-month, interpolating between each mid-month value. To get the test demand estimate, we fit a parameter ρ_S that represents to likelihood of non-infected individual seeking a test compared to a symptomatically infected individual.

$$Test\,pop_i = \rho_{si} * \left(S_i + \textstyle\sum_i^j (A_{ij} + AV_{ij} + P_{ij} + PV_{ij} + E_{ij} + EV_{ij})\right) + \textstyle\sum_i^j (I_{ij} + IV_{ij})$$

$$d_i = \frac{Tests_i}{Test\,pop_i}$$

The forces of infection (λ_{ij}), representing the risk of the susceptible individuals by age to acquire infection (transition from susceptible to exposed), are differentiated by age of the susceptible individual, a contact matrix (proportion of contacts with each age group), infection and treatment status (asymptomatic, pre-symptomatic, symptomatic, diagnosed and hospitalized cases) of the infected contacts, and the time-dependent reduction of transmission due to physical distancing measures (work from home, closing non-essential businesses, banning large gathering, etc.) applied in the area (scaled up starting March 8 and fully taking effect March 29) and later relaxed during the reopening after May 15 to values that are fit monthly until Oct 31st, 2020.

$$\begin{aligned}\lambda_{ij} = \sum_{k=1}^{4} c_{ik}\,(1 - R_{sd}(t)) \Bigg[\sum_i^j \sum_i (\beta_a A_{jk} + \beta_p P_{jk} + \beta_s I_{jk} + \beta_d D_{jk} + \beta_{da} DA_{jk}) \\ + (1 - VE_{INF}) \left(\sum_i^j \sum_i (\beta_a AV_{jk} + \beta_p PV_{jk} + \beta_s IV_{jk} + \beta_d DV_{jk} + \beta_{da} DAV_{jk}) \right) \Bigg] / N_i \\ + c_{ik}\beta_h \sum_i^j (H_{jk} + HV_{jk}) / N_i\end{aligned}$$

where β_a, β_p, β_s, β_d, β_h are the transmission rates from contacts with asymptomatic, pre-symptomatic, symptomatic, diagnosed, and hospitalized infections (before the start of COVID measures at t= δ_1), c_{ik} is contact matrix (proportion of the contact with other age groups), N_i is population size by age, VE_{SUSC} is vaccine efficacy in reducing the acquisition risk (reduction of susceptibility), VE_{INF} is vaccine efficacy in reducing the transmission risk (reduction of infectiousness).

$R_{sd}(t)$ is the reduction of transmission due to physical distancing and other preventive measures which is applied uniformly to all age groups. It is scaled up linearly from 0 to R_{sd}^{max} between t= δ_1 and t= δ_2) at the beginning of the lockdown in March. Later it is calibrated monthly to match the King County epidemic through December of 2020 and then controlled dynamically based on the bi-weekly case rates per 100k of the population. For age groups 1-3 the highest value of R_{sd}^{max} is 0.6 (i.e. interactions at 40% of pre-COVID levels) and the lowest allowed is 0.3 (social interactions at 70% of pre-COVID levels). These limits are each 0.2 higher for the oldest age group. The triggers for increasing or decreasing social distancing levels are given in the parameter table.

King County model calibration. The model is calibrated to 3 "targets" based on local data (**Supplementary Figures S2, S3**), namely: the age-wise number of confirmed daily cases, daily hospital admissions, and daily deaths reported in King County over time since the start of the epidemic outbreak through December 31st, 2020. We used the BFGS optimization algorithm to estimate the best parameter values for the time period being fitted. We defined thresholds for each parameter and proceeded with the best set reported by the routine selected by the

optimization algorithm. Calibration was divided into multiple periods. The first was from the start of the epidemic through the initial lockdown period ending in early May. Subsequent fits were by month, but shared the initial fits for start date, β_* (overall infectivity) and β_d (adjustment to infectivity for diagnosed individuals).

Intra-host model of SARS-CoV-2 kinetics. This model is used in the paper (*section 3*.8) to predict the impact of lowering viral load on vaccine efficacy against transmissibility given infection (VE_{INF}) assumes SARS-CoV-2 (*V*) infects susceptible cells (*S*) at rate β producing infected cells (I) that then generate new virus at a per-capita rate π. In the model, the death of infected cells is mediated by (1) the innate responses (δI^k) which is dependent on the infected cell density and the exponent k, and (2) the acquired immune responses by SARS-CoV-2-specific effector cells (E). The acquired responses are non-linear. More details on the model can be found in the ref (2).

The model is expressed as a system of ordinary differential equations described in the Methods and characterized in Sup fig 1. The initial conditions for the model were assumed as $S(0) = 10^7$ cells/mL, $I(0) = 1$ cells/mL, $V(0) = \frac{\pi I(0)}{\gamma}$ copies/mL. E_0 is the number of cytolytic immune cells and is varied between simulations to approximate different vaccine efficacies. For simulations, we sampled parameter values from a nonlinear mixed-effect model, with the following fixed effects and standard deviation of the random effects (in parenthesis): $Log_{10}\beta$: -7.23 (0.2) virions^{-1} day^{-1}; δ: 3.13 (0.02) day^{-1} cells^{-k}; k: 0.08 (0.02); Log10(π): 2.59 (0.05) day^{-1}; m: 3.21 (0.33) days^{-1}cells^{-1}; Log10(ω): -4.55 (0.01) days^{-1}cells^{-1}. These parameter values were obtained by fitting to serial viral load data in (2) using nonlinear mixed effects modeling. We further fixed $m = 0.01$ days^{-1}cells^{-1} and $\gamma = 15$ day^{-1}

Intra-host transmission model. To estimate SARS-CoV-2 infectiousness $P_t[V(t)]$ in *section 3.*8 we employed the function, $P_t[V(t)] = \frac{V(t)^\alpha}{\lambda^\alpha + V(t)^\alpha}$. Here, $V(t)$ is the viral load of the transmitter obtained from our previously proposed within host model and estimates (*2*). λ is the infectivity parameter that represents the viral load that corresponds to 50% infectiousness and 50% contagiousness, and α is the Hill coefficient that controls the slope of the dose-response curve. Our transmission model assumes that only some contacts of an infected individual with viral load dependent infectiousness are physically exposed to the virus (defined as exposure contacts), that only some exposure contacts have virus passaged to their airways (contagiousness) and that only some exposed contacts with virus in their airways become secondarily infected (successful secondary infection). Contagiousness and infectiousness are then treated as viral load dependent multiplicative probabilities with transmission risk for a single exposure contact being the product. Contagiousness is considered to be viral load dependent based on the concept that a transmitter's dispersal cloud of virus is more likely to prove contagious at higher viral load, which is entirely separate from viral infectivity within the airway once a virus contacts the surface of susceptible cells. Details can be found in (*3, 4*).

We assumed that the total exposed contacts within a time step (η_{Δ_t}) is gamma distributed, i.e. $\eta_{\Delta_t} \sim \Gamma\left(\frac{\theta}{\rho}, \rho\right)\Delta_t$, using the average daily contact rates (θ) and the dispersion parameter (ρ). To obtain the true number of exposure contacts with airway exposure to virus, we multiply the contagiousness of the transmitter by the total exposed contacts within a time step (i.e., $\zeta_t = \eta_{\Delta_t} P_t$). Transmissions within a time step are simulated stochastically using time-dependent viral load to determine infectiousness (P_t). Successful transmission is modelled stochastically by drawing a random uniform variable ($U(0,1)$) and comparing it with

infectiousness of the transmitter. In the case of successful transmission, the number of secondary infections within that time step (T_{Δ_t}) is obtained by the product of the infectiousness (P_t) and the number of exposure contacts drawn from the gamma distribution (ζ_t). In other words, the number of secondary infections for a time step is $T_{\Delta_t} = \mathrm{Ber}(P_t) P_t \eta_{\Delta_t}$. We obtain the number of secondary infections from a transmitter on a daily basis noting that viral load, and subsequent risk, does not change substantially within a day. We then summed up the number of secondary infections over 30 days since the time of exposure to obtain the individual reproduction number, i.e. $R_0 = \sum_{\Delta_t} T_{\Delta_t}$.

We further assume that upon successful infection, it takes τ days for the virus to move within-host, reach the infection site and produce the first infected cell. To calculate serial interval (time between the onset of symptoms of transmitter and secondarily infected person), we sample the incubation period in the transmitter and in the secondarily infected person from a gamma distribution (*5, 6*). In cases in which symptom onset in the newly infected person precedes symptom onset in the transmitter, the serial interval is negative; otherwise, serial interval is non-negative.

The model was fit to distributions of individual R0 (secondary transmissions per person) and serial interval as previously described (*7-11*). We then arrived at parameter estimates for λ, τ, α and θ and identified that a skewed distribution of daily exposure contacts explains the virus super-spreader property. This model was used to obtain baseline levels of secondary transmission for simulated placebo recipients.

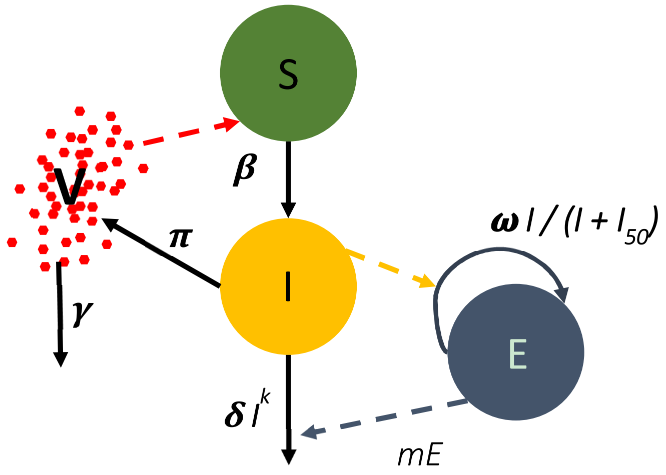

Supplementary Figure S1. Intra-host mathematical model schematic. S=susceptible cells, I = infected cells, E = effector immune cells, V = virus, β=infectivity, π=viral production rate, δI^k=density dependent death rate, mE=effector cell killing rate, $\omega I/(I+I_{50})$=effector cell killing rate, and γ=viral clearance rate.

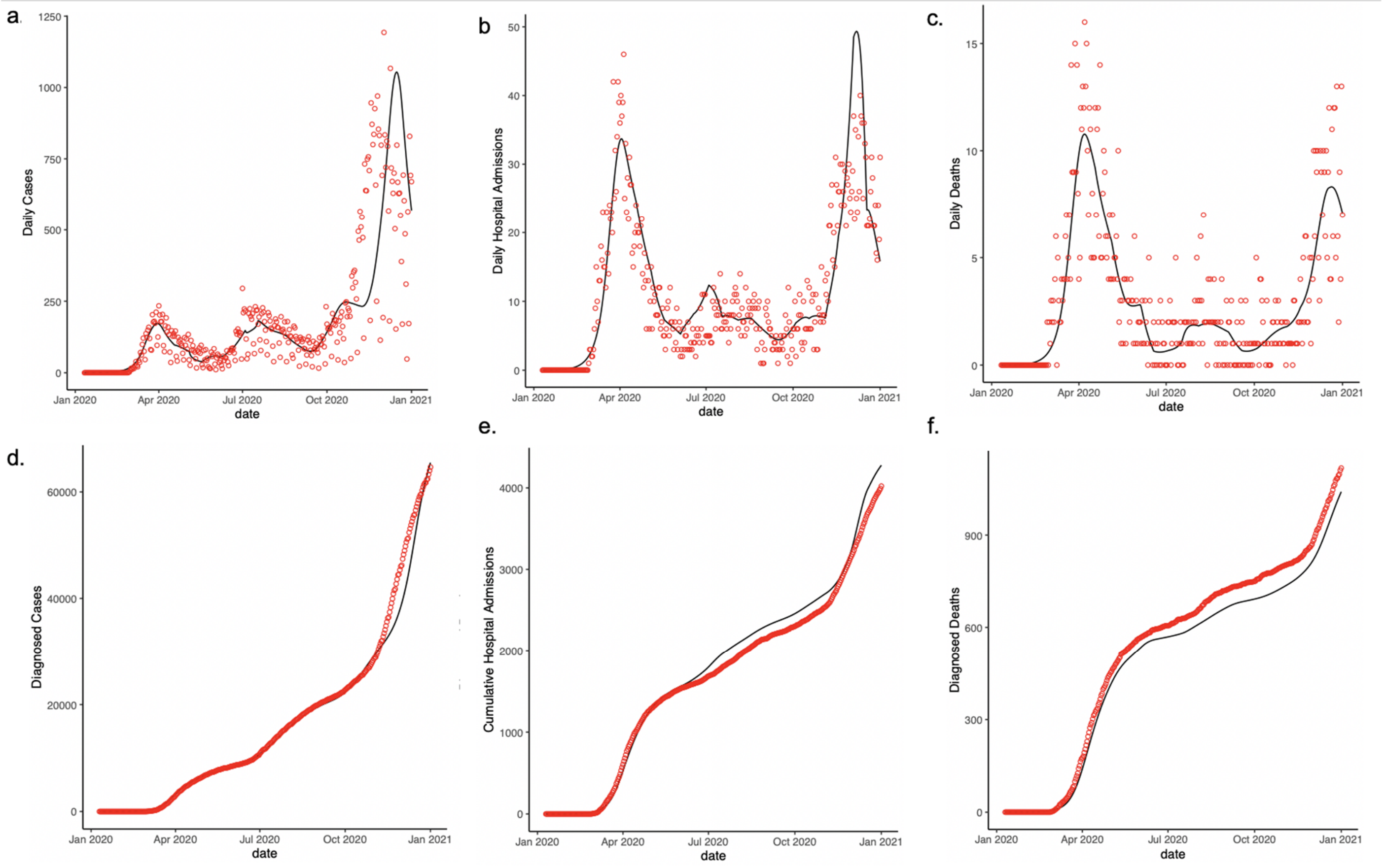

Supplementary Figure S2. Calibration of a SARS-CoV-2 transmission model in King County, Washington between January 1, 2020 and January 1, 2021. Model fit is to **a.** daily cases, **b.** daily hospitalizations, **c.** daily deaths, **d.** cumulative cases, **e.** cumulative hospitalizations, and **f.** cumulative deaths through the end of October 2021.

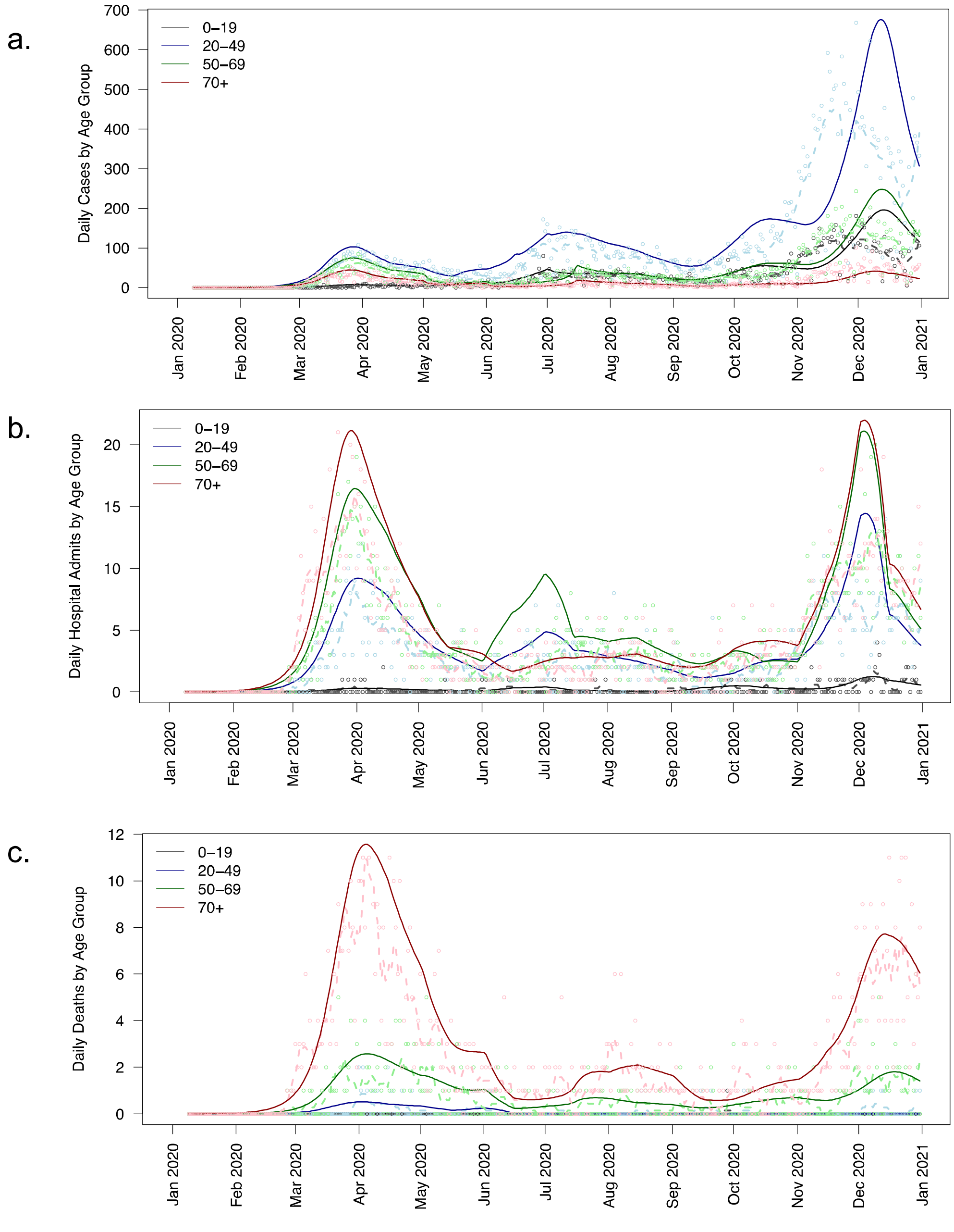

Supplementary Figure S3. Calibration of a SARS-CoV-2 transmission model in King County, Washington between January 1, 2020 and January 1, 2021. Model fit is to **a.** age-stratified cases, **b.** age-stratified hospitalizations and **c.** age-stratified deaths.

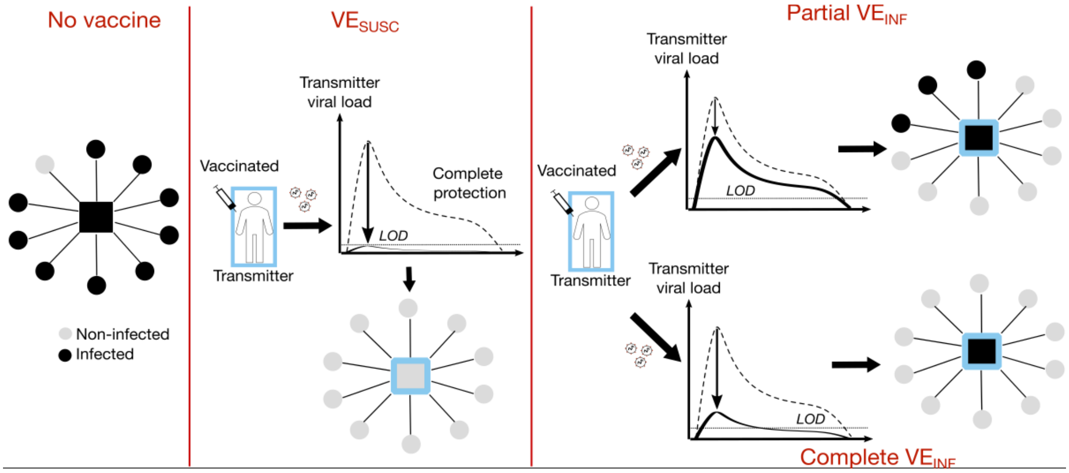

Supplementary Figure S4. Conceptual basis for reduction in viral load lowering transmission.

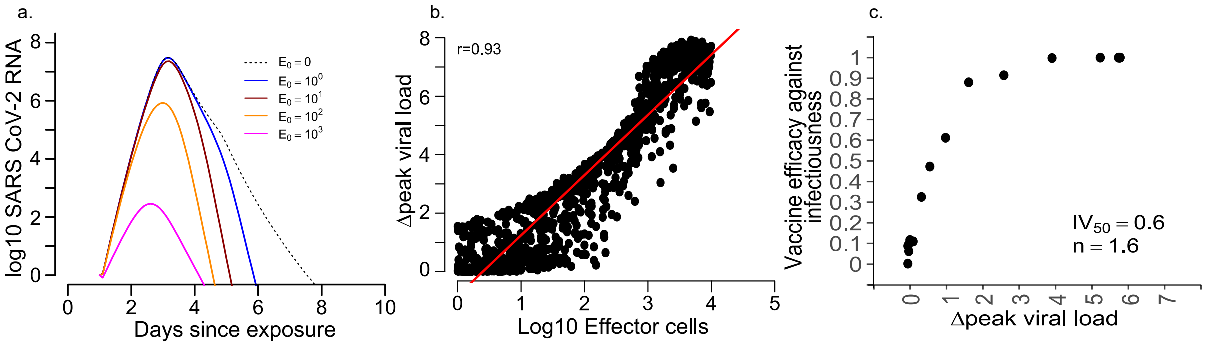

Supplementary Figure S5. Small reduction in peak viral load due to vaccinations would translate to significant VE_{INF}. a. Simulated virologic trajectories with higher imputed initial number of vaccine-generated tissue-resident immune cells (E_0) demonstrate lower peak viral loads. b. Varying number of tissue-resident immune effector cells generated by a vaccine (x-axis) predicts peak viral load (y-axis) in individual infection simulations each denoted with a dot. The red line indicates a correlation line. c. Reduction in viral load (x-axis) predicts VE_{INF} (y-axis) in vaccine simulations. Each black dot is a simulation of 1000 vaccine recipients given a vaccine which generates a fixed E0 versus 1000 placebo recipients. VE_{INF}=50% is achieved with a 0.6 $\log_{10}$ reduction in peak viral load. VE_{INF}=90% is achieved with a 2.5 $\log_{10}$ reduction in peak viral load. The relationship between change in peak viral load (x) and VE_{INF} is captured with the formula: $VE_{INF} = (\log_{10} x)^{1.6} / (IV50)^{1.6} + (\log_{10} x)^{1.6}$ where IV50=0.6.

Supplementary Table S1. Parameters and ranges used in the analysis. (Fixed in black, Calibration in red)

Parameter	Description	Values and ranges	Type
γ_1	Progression rates from exposed (E) to infectious (A or P) (latent time)$^{-1}$	(3 days)$^{-1}$	Fixed
γ_2	Progression rates from pre-symptomatic (P) to symptomatic (I) (pre-symptomatic time)$^{-1}$	(2 days)$^{-1}$	Fixed
p_i	Proportion of the infections which become symptomatic by age	80%	Fixed
$\rho_S(i, j)$	Relative likelihood of susceptibles seeking Testing by age (i)	$\rho_S(i, j)$=0.1-10%	Fit Monthly (see table S2)
id	Symptomatic infectiousness duration	7 days	Fixed
β_i	Daily transmission from infected from asymptomatic, pre-symptomatic, symptomatic, diagnosed and hospitalized groups in absence of COVID measures	$=\beta s*(1, \beta_p, 1, \beta_d, 0)$<br>$\beta_p$ calculated to get 44% pre-sympt. transmission<br>$\beta_s = R0/(\beta p/\gamma_2 + id) =$ 0.2 (R0 = 2.2-4)<br>β_d calibrated during lockdown (0.6)	Calibrated
R_{sd}^{min}	Minimum SD during dynamic SD periods	0.3, 0.5 for 70+	Fixed
R_{sd}^{max}	Maximum SD during dynamic SD periods	0.6, 0.8 for 70+	Fixed
SDtighten	Trigger for tightening SD (bi-weekly cases per 100k)	350	Fixed
SDrelax	Trigger for relaxing SD (bi-weekly cases per 100k)	100	Fixed
SDrelax rate	Rate for relaxing SD (bi-weekly percentage to min)	10%	Fixed
R_{sd}^{fit}	Reduction of transmission due to	10%-90%	Fit Monthly

	social distancing (scaled up linearly between t= δ_1 and t= δ_2)		(see table S2)
δ_0	Number of days between the start of the simulation (day 0) and the 1st diagnosed case from data (Feb 28)	49 (45-55)	Calibrated
[$\delta 1$, $\delta 2$]	Period of scaling up COVID measures	March 8-29	Fixed
[δ_3, δ_4]	Reopening period	May 15- July 15	Fixed
hf_i	Proportion of diagnosed cases requiring hospitalization		Monthly Average from DOH data
h_i	Hospitalization rate among severe cases by age	h_1=0.005-0.1, h_2=0.01-0.2, h_3=0.01-0.3, h_4=0.01-0.5	Fit Monthly (see table S2)
h_i^*	Hospitalization rate among symptomatic and diagnosed cases	$= (hf_i)h_i$	Calculated
r_1	Recovery rate of asymptomatic cases	1/id	Fixed
r_i^*	Recovery rate of the symptomatic and diagnosed cases	$= (1 - hfi) * r_1$	Calculated
r_3	Recovery rate of the hospitalized cases	1/14	Fixed
hd	Time from hospitalization to death before and after April 15th	11.2 days/ 20 days	fixed
nhd	Time from diagnosis to death based on non-hospitalized COVID deaths	24 days	fixed
f_i	Fatality rate by age among hospitalized (overall mortality when hospitalized/time to death)	$=cfr_i/(hf_i)/hd$	Fit Monthly (see table S2)
nf_i	Fatality rate by age among symptomatically infected, not hospitalized (overall mortality when hospitalized/time to death)		

Supplementary Table S2. Monthly Parameter Fits. The columns represent the distribution of each parameter across the 4 age groups for each calibrated month:

Parameter	Age(y)	Lockdown	May	June	July	Aug	Sept	Oct*	Nov*	Dec*
ρ_{Si}*	**0-19**	0.018	0.007	0.004	0.012	0.01	0.009	0.009	0.009	0.009
**(held constant**	**20-49**	0.004	0.007	0.003	0.006	0.006	0.007	0.007	0.007	0.007
**during Oct-Dec)**	**50-69**	0.003	0.017	0.093	0.014	0.011	0.011	0.011	0.011	0.011
	**70+**	0.001	0.009	0.153	0.02	0.025	0.075	0.075	0.075	0.075
$R^{fit}_{sd\,i}$	**0-19**	0.5	0.52	0.13	0.4	0.42	0.11	0.6	0.1	0.6
	**20-49**	0.7	0.6	0.3	0.54	0.57	0.31	0.27	0.1	0.6
	**50-69**	0.7	0.6	0.44	0.55	0.56	0.46	0.6	0.1	0.6
	**70+**	0.9	0.8	0.77	0.79	0.79	0.77	0.76	0.1	0.8
$\mathbf{h_i}$	**0-19**	0.014	0.015	0.079	0.027	0.02	0.076	0.044	0.076	0.032
	**20-49**	0.05	0.053	0.16	0.085	0.09	0.067	0.094	0.169	0.042
	**50-69**	0.068	0.045	0.17	0.056	0.07	0.067	0.036	0.118	0.026
	**70+**	0.106	0.071	0.038	0.032	0.04	0.045	0.032	0.088	0.024
$\mathbf{cfr_i}$	**0-19**	0	0	0	0	0	0	0	0	0
	**20-49**	0.001	0.001	0	0	0	0	0	0	0
	**50-69**	0.017	0.026	0.006	0.005	0.003	0.005	0.003	0.002	0.002
	**70+**	0.268	0.249	0.121	0.105	0.088	0.067	0.119	0.16	0.149

Supplementary Table S3. Monthly Hospital Admission Fractions from the Washington Department of Health. The rows represent the distribution of each input across the 4 age groups for each input period:

Period\Input	Hosp fraction (hf): 0-19, 20-49, 50-69, 70+
**Lockdown (Feb/Mar)**	0.017, 0.07, 0.2, 0.58
**April**	0.018, 0.093, 0.234, 0.53
**May**	0.015, 0.068, 0.198, 0.43
**June**	0.011, 0.03, 0.126, 0.375
**July**	0.006, 0.029, 0.12, 0.315
**August**	0.0026, 0.027, 0.12, 0.364
**September**	0.01, 0.025, 0.102, 0.333
**October**	0.008, 0.015, 0.082, 0.385
**November**	0.005, 0.018, 0.074, 0.296
**December**	0.008, 0.023, 0.088, 0.296

Supplementary Table S4. Contact matrix. The columns represent the distribution of contacts of a person from given age group across all age groups:

**Proportion contacts with**	**0-19 y**	**20-49 y**	**50-69 y**	**70+ y**
**0-19 y**	0.56	0.24	0.15	0.18
**20-49 y**	0.34	0.57	0.49	0.34
**50-69 y**	0.08	0.16	0.29	0.28
**70+ y**	0.01	0.03	0.07	0.20

Supplementary Table S5. King County age pyramid based on data from 2017

**Proportion of the population**	**0-19 y**	**20-49 y**	**50-69 y**	**70+ y**
	22.93%	45.52%	23.50%	8.05%

References

1. C. Bracis *et al.*, Widespread testing, case isolation and contact tracing may allow safe school reopening with continued moderate physical distancing: A modeling analysis of King County, WA data. *Infectious Disease Modelling* **6**, 24-35 (2021).
2. A. Goyal, E. F. Cardozo-Ojeda, J. T. Schiffer, Potency and timing of antiviral therapy as determinants of duration of SARS-CoV-2 shedding and intensity of inflammatory response. *Sci Adv* **6**, (2020).
3. A. Goyal, D. B. Reeves, E. F. Cardozo-Ojeda, J. T. Schiffer, B. T. Mayer, Viral load and contact heterogeneity predict SARS-CoV-2 transmission and super-spreading events. *Elife* **10**, (2021).
4. A. Goyal, D. B. Reeves, E. F. Cardozo Ojeda, B. T. Mayer, J. T. Schiffer, Slight reduction in SARS-CoV-2 exposure viral load due to masking results in a significant reduction in transmission with widespread implementation. *medRxiv*, 2020.2009.2013.20193508 (2020).
5. T. Ganyani *et al.*, Estimating the generation interval for coronavirus disease (COVID-19) based on symptom onset data, March 2020. *Euro Surveill* **25**, (2020).
6. S. A. Lauer *et al.*, The Incubation Period of Coronavirus Disease 2019 (COVID-19) From Publicly Reported Confirmed Cases: Estimation and Application. *Ann Intern Med* **172**, 577-582 (2020).
7. Q. Bi *et al.*, Epidemiology and transmission of COVID-19 in 391 cases and 1286 of their close contacts in Shenzhen, China: a retrospective cohort study. *Lancet Infect Dis*, (2020).
8. Y. Zhang, Y. Li, L. Wang, M. Li, X. Zhou, Evaluating Transmission Heterogeneity and Super-Spreading Event of COVID-19 in a Metropolis of China. *Int J Environ Res Public Health* **17**, (2020).
9. A. Dillon *et al.*, Clustering and superspreading potential of severe acute respiratory syndrome coronavirus 2 (SARS-CoV-2) infections in Hong Kong. *PREPRINT (Version 1) available at Research Square*, (2020).
10. A. Endo, Centre for the Mathematical Modelling of Infectious Diseases COVID-19 Working Group, S. Abbott, A. Kucharski, S. Funk, Estimating the overdispersion in COVID-19 transmission using outbreak sizes outside China. *Wellcome Open Res* **5**, (2020).
11. Z. Du *et al.*, Serial Interval of COVID-19 among Publicly Reported Confirmed Cases. *Emerg Infect Dis* **26**, 1341-1343 (2020).
